# Comparison of glottic views and intubation times in the supine and 25 degree back-up positions

**DOI:** 10.1186/s12871-016-0280-4

**Published:** 2016-11-16

**Authors:** Raj M Reddy, Manish Adke, Pranava Patil, Irina Kosheleva, Saxon Ridley, on behalf of the Anaesthetic Department at Glan Clwyd Hospital, S Agarwal, S Ajam, A Bhatnager, L Bandara, C Beaton, G Bugelli, S Burgess, J Butler, S Chugani, D Cliciovans, D Counsell, J Dougherty, L Dumby, A Evans, C Fulton, S Ganesh, C Goodman, L Griffiths, I Gyorimolnar, Z Hajat, P Hamer, E Hosking, S Hugo, A Idries, A Jacob, P Jirasck, D John, N Juganaru, N Kelly, A Khalil, I Khan, M Khater, P Kucharski, G Leslie, M Liutkus, V Machineni, M Marimuthu, P Michael, A Moss, G Mula, N Murphy, D Pausan, R Pugh, M Quarmby, K Rafferty, A Rajpal, S Raza, D Redfern, N Roy, A Sampath, R Scale, S Shenoy, R Shobha, A Slater, Y Soon, A Sultanpori, L Tee, D Thaker, U Ramesh, C Variu, C Von Sass, D Zabauski

**Affiliations:** Anaesthetic Department, Glan Clwyd Hospital, Betsi Cadwaladr University Health Board, Sarn Lane, Rhyl, LL18 5UJ UK

**Keywords:** Intubation, Back-up position, Glottis, View

## Abstract

**Background:**

We explored whether positioning patients in a 25° back-up sniffing position improved glottic views and ease of intubation.

**Methods:**

In the first part of the study, patients were intubated in the standard supine sniffing position. In the second part, the back of the operating table was raised 25° from the horizontal by flexion of the torso at the hips while maintaining the sniffing position. The best view obtained during laryngoscopy was assessed using the Cormack and Lehane classification and Percentage of Glottic Opening (POGO) score. The number of attempts at both laryngoscopy and tracheal intubation, together with the use of ancillary equipment and manoeuvres were recorded. The ease of intubation was indirectly assessed by recording the time interval between beginning of laryngoscopy and insertion of the tracheal tube.

**Results:**

Seven hundred eighty one unselected surgical patients scheduled for non-emergency surgery were included. In the back-up position, ancillary laryngeal manoeuvres, which included cricoid pressure, backwards upwards rightward pressure and external laryngeal manipulation, were required less frequently (19.6 % versus 24.6 %, *p* = 0.004). The time from beginning of laryngoscopy to insertion of the tracheal tube was 14 % shorter (median time 24 versus 28 s, *p* = 0.031) in the back-up position. There was no significant difference in glottic views.

**Conclusions:**

The 25° back-up position improved the ease of intubation as judged by the need for fewer ancillary manoeuvres and shorter time for intubation.

**Trial registration:**

ClinicalTrials.gov Identifier: NCT02934347 registered retrospectively on 14th Oct 2016.

**Electronic supplementary material:**

The online version of this article (doi:10.1186/s12871-016-0280-4) contains supplementary material, which is available to authorized users.

## Background

The horizontal supine sniffing position has traditionally been considered the optimal head position for direct laryngoscopy and is preferred by most anaesthetists. Although contentious [1], neck flexion theoretically aligns the pharyngeal and laryngeal axes, and head extension at the atlanto-occipital joint aligns the oral axis with these two axes allowing the line of sight to fall on the glottis [2]. Magnetic resonance images through the external auditory meatus and midline confirms greater elevation of the nasopharynx relative to the glottis in the sniffing position [3] so supporting the classical position. However in conjunction with alignment of the relevant anatomical structures, it is important that the intubating anaesthetist’s line of sight falls easily and comfortably on the glottic aperture.

The back-up position achieved by flexion of the torso at the hips was described by Chevalier Jackson almost a century ago [4]; such a position may improve the line of sight for anaesthetist standing behind the patient’s head. In a 25° back-up position less force is required to elevate and move the tongue and other tissues out of the line of sight [5]. Also the quality of the laryngeal views improves and anaesthetists feel less discomfort during tracheal intubation with operating table horizontal but raised level to the anaesthetist’s xiphoid and nipple [6]. This avoids the required stooping by the anaesthetist to acquire a view of the larynx and could be achieved by a 25° back-up position.

In clinical practice, the back-up position has been successfully used in obese patients [7, 8] and shown to improve efficiency of pre-oxygenation [9] and so increase the duration of ‘safe’ apnoea during intubation [10]. Lebowitz [11] reported that the use of a ramp provided significantly better or equal laryngoscopic views, relative to those with the supine sniffing position in 189 adults. As a result we hypothesized that if the back-up position aids glottic views in situations where intubation is anticipated to be difficult, then using such a position routinely may be advantageous if it helps bring the line of sight onto the glottis more easily or more comfortably.

Therefore the aim of this study was to test whether a 25° back-up position improves laryngeal views and makes intubation easier compared to the standard horizontal position.

## Methods

The study outline was reviewed by North Wales Research Ethics Committee of the Betsi Cadwaladr University Health Board who deemed that the observation of routine established anaesthetic techniques should not be classified as research and therefore further consideration by the Ethics Committee and individual patient consent was not required (Committee reference No SL 24, 2011). The project was registered with the Health Board’s Research and Development Department.

The study had two parts. Part 1 was an observational period of standard practice to ascertain the distribution of glottic views and ease of intubation with the patient in the horizontal sniffing position. Part 2 was identical except that the patients had their anaesthesia induced and tracheas intubated in a 25° back-up position achieved by flexion of the operating table at the hips.

Adult patients who required intubation as part of their routine anaesthesia were studied. However, the following groups of patients were excluded:

1. Patients less than 18 years old,
2. Patients recognised to have difficult airways where an alternative method of intubation (e.g. fibre optic) was the method of choice,
3. Patients undergoing emergency surgery where patient positioning and data collection might cause delay (e.g. exsanguinating patients) or where the supine position is not optimal (e.g. brisk bleeding into the upper airway),
4. Patients requiring rapid sequence induction of anaesthesia.

The data collection form (see Additional file 1) asked the intubating anaesthetist to confirm that:

1. all patients were in the standard sniffing position with a head ring or non-compressible pillow so that a line from the sternal notch to external auditory meatus was horizontal.
2. in Part 2 only, the back of the operating table was raised 25° from the horizontal (Fig. 1) while maintaining the classical sniffing position.

**Fig. 1:**
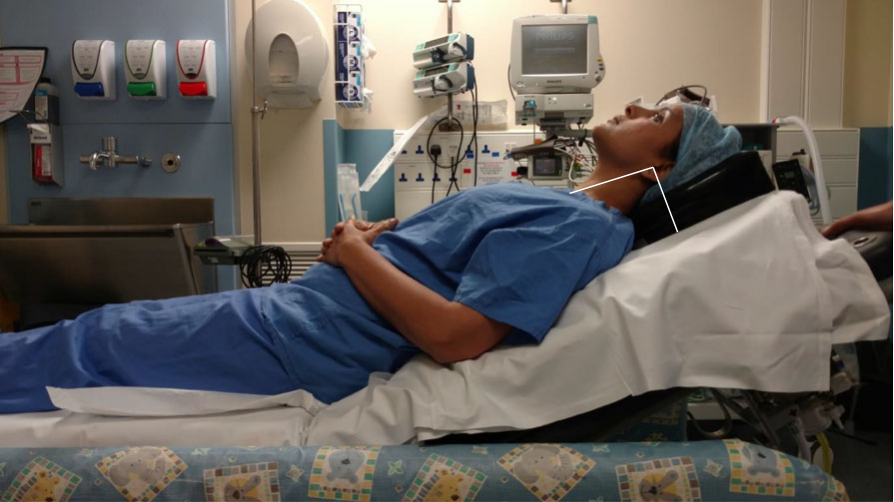
The 25° back-up position. Note flexion of the torso at the hips and the line joining external auditory meatus to the sternal notch being parallel with the operating table
3. direct laryngoscopy was performed using an adult Macintosh blade (size 3 or 4) after suppression of all twitches on a train-of-four monitor.

The choice of anaesthetic type and agents was left to the discretion of the anaesthetist. All members of the Anaesthetic Department at Glan Clwyd Hospital were invited to participate. The project and in particular the patient positioning and data collection requirements were presented at a Departmental Meeting.

Patient demographics (i.e. age, sex, height and weight or body mass index) and drugs used at induction were recorded. The best view obtained during laryngoscopy was assessed using the Cormack and Lehane classification [12] and Percentage of Glottic Opening (POGO) score [13] by the anaesthetist performing the laryngoscopy. Illustrations of the Cormack and Lehane views and POGO score were included on the data collection form as an ‘*aide memoire*’. The number of attempts at both laryngoscopy and tracheal intubation were recorded. The use of ancillary equipment (e.g. bougie, alternative laryngoscope blades) and manoeuvres (e.g. laryngeal manipulation) were recorded but applied at the intubating anaesthetist’s discretion. The time between the beginning of laryngoscopy and detection of CO_2_ on the end-tidal CO_2_ monitor after the successful placement of the tracheal tube was recorded by either the intubating anaesthetist or other members of the anaesthetic team.

Descriptive statistics for normally distributed data are presented as means and standard deviations (SD) while skewed data is summarised as medians and interquartile ranges (IQR). Mann–Whitney U tests were used to explore differences in non-normally distributed data while Chi-squared tests or Chi-squared tests for trend explored difference in frequencies. Assuming an incidence of Cormack and Lehane Grade I views to be 95 % and that a 4 % increase in these views can be achieved by a 25° back-up position, 325 patients in both parts would be required to have a power of 0.8 and an alpha error of 0.05. Statistical analysis was performed using Minitab (version 15). A p value of less than 0.05 was considered significant.

## Results

The study enrolled a total of 781 unselected patients (374 in Part 1 and 407 in Part 2) between December 2011 and November 2014 representing an average enrolment rate of 22 patients per month. Sixty-eight members of the Anaesthetic Department recorded data on glottic views; experience varied from consultant anaesthetists with over 20 years experience to trainees with just over 1 year. There was no significant difference between numbers of training versus senior grades contributing to either part of the study (Table 1). Most members recorded data on fewer than 10 patients; the first four authors were responsible for enrolling 372 patients.

**Table 1:** Patient, intubating anaesthetist and surgical details

	Supine (Part 1) (*n* = 374)	Back-up (part 2) (*n* = 407)
Age, yrs (mean, SD)	57.4 (17.6)	55.8 (16.9)
Male: Female (n)	186:188	188:218
Body Mass Index (mean, SD)	28.5 (5.5)	28.0 (5.8)
Intubating anaesthetist (n, %)		
Senior grades	272 (73)	264 (65)
Training grades	101 (27)	143 (35)
Surgery, n (%)		
General	248 (66.3)	233 (57.2)
Gynaecology	44 (11.8)	24 (5.9)
Urology	29 (7.7)	19 (4.7)
ENT	24 (6.4)	43 (10.6)
Ophthalmology	0	1 (0.2)
Orthopaedics	12 (3.2)	3 (0.7)
Facio-Maxillary	10 (2.7)	62 (15.2)
Trauma	4 (1.1)	18 (4.4)
Vascular	2 (0.6)	4 (1.0)
Not recorded	1 (0.2)	0

The demographic details of the patients are summarised in Table 1 while details of the anaesthetic agents used are given in Table 2. There were no differences between the two groups with respect to their age, sex and body mass index. The range of surgical procedures was dissimilar with the greatest imbalances being within facio-maxillary and gynaecology surgery (Chi-squared = 51.4, df =5, *p* < 0.001). The distribution of anaesthetic agents was similar in both groups except that atracurium was more frequently used in the second phase (Part 1, atracurium 52.9 % versus rocuronium 44.1 %; Part 2, atracurium 59.0 % versus rocuronium 36.1 %, Chi-squared = 9.76, df = 3, *p* = 0.021). There were no reports of difficulty with mask ventilation in any patients.

**Table 2:** Anaesthetic agents used

		Supine (Part 1)	Back-up (part 2)
		*n* (%)	*n* (%)
Induction	Propofol	373 (99.7)	404 (98.3)
	Sevoflurane	0	1 (0.2)
	Not recorded	1 (0.3)	2 (0.5)
Analgesia	Fentanyl	252 (67.4)	246 (60.4)
	Remifentanil	113 (30.2)	153 (37.6)
	Alfentanil	5 (1.3)	5 (1.2)
	Not recorded	4 (1.1)	3 (0.7)
Neuromuscular blocking agent	Atracurium	198 (52.9)	240 (59.0)
	Rocuronium	165 (44.1)	147 (36.1)
	Suxamethonium	8 (2.1)	8 (2.0)
	Vecuronium	1 (0.3)	8 (2.0)
	Mivacurium	0	1 (0.2)
	Not recorded	2 (0.6)	3 (0.7)

With regard to intubation, 90.5 % patients in both parts of the study were intubated successfully at the first direct laryngoscopy and first attempt at intubation (Table 3). The use of ancillary equipment did not differ between the groups. Although small in number, more nasal tubes were inserted in the second part of the study (2.5 % versus 0.6 %, Chi-squared = 4.95, df = 1, *p* = 0.025). In the back-up position, ancillary laryngeal manoeuvres (which included cricoid pressure, backwards upwards rightward pressure and external laryngeal manipulation) were required less frequently (19.6 % versus 24.6 %, Chi-squared = 13.14, df = 3, *p* = 0.004).

**Table 3:** Intubations

		Supine (Part 1) (*n* = 374)	Back-up (Part 2) (*n* = 407)
Type, n (%)	Oral	369 (98.7)	387 (95.0)
	Nasal	2 (0.6)	10 (2.5)
	Not recorded	3 (0.7)	10 (2.5)
Laryngoscopy attempts, n (%)	1	340 (90.9)	371 (91.2)
	2	25 (6.7)	27 (6.6)
	>2	5 (1.3)	3 (0.7)
	Not recorded	4 (1.1)	6 (1.5)
Intubation attempts, n (%)	1	346 (92.5)	378 (92.9)
	2	22 (5.9)	18 (4.4)
	>2	0	1 (0.2)
	Not recorded	6 (1.6)	10 (2.4)
Ancillary equipment used, n (%)	Bougie	58 (15.5)	61 (15.0)
	Other (McCoy, airway, Magill forceps, stylet)	3 (0.7)	4 (1.0)
	Recorded as ‘Not used’	308 (82.4)	341 (83.7)
	Not recorded	5 (1.3)	1 (0.2)
Ancillary manoeuvres, n (%)	Cricoid pressure	19 (5.1)	2 (0.4)
	Backwards, upwards, rightward pressure	34 (9.1)	25 (6.1)
	External laryngeal manipulation	39 (10.4)	53 (13.0)
	Recorded as ‘Not used’	277 (74.1)	326 (80.1)
	Not recorded	5 (1.3)	1 (0.2)

Overall, the time from beginning of laryngoscopy to insertion of the tracheal tube was significantly shorter when performed by senior grades as opposed to trainees (median time 24 versus 28 s, *p* = 0.001). In the second part of the study, the time from the beginning of laryngoscopy to tube insertion was significantly shorter for both training and senior grades (median time 24 versus 28 s, *p* = 0.031) (Table 4).

**Table 4:** Time from beginning of laryngoscopy to insertion of the tracheal tube in seconds (median and IQR)

Grade of intubating anaesthetist	Supine	Back-up
Senior	26 (17.25 to 40)	21 (16 to 36)
Training	30 (22 to 47.50)	27 (18.75 to 42)
Combined senior and training grades	28 (18 to 42)	24 (16 to 39)
Senior	24 (16 to 40)	
Training	28 (20 to 45)	

**Fig. 2:**
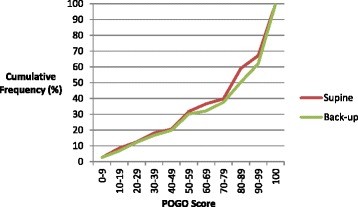
Distribution of POGO scores shown as cumulative frequency curves (Supine *red*, back-up *green*)

**Fig. 3:**
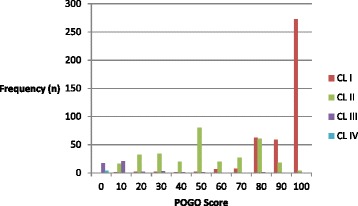
Distribution of POGO scores grouped by Cormack and Lehane Grades (Grade I *red*, Grade II *green*, Grade III *purple*, Grade IV *blue*)

There was no significant difference in glottic views when assessed by the Cormack and Lehane Grades (Table 5) or POGO score (Fig. 2) in either the part of the study. The views recorded were not dependent upon training status. In total, the distribution of Cormack and Lehane views were Grade I 53.1 %, Grade II 40.3 %, Grade III 6.0 % and Grade IV 0.5 %. The median POGO score for all patients was 80 (IQR 50 to 100). The distribution of Cormack and Lehane Grades in each decile of POGO score is shown in Fig. 3.

**Table 5:** Glottic views

Cormack and Lehane Grades	Supine (Part 1) (*n* = 374) n (%)	Back-up (Part 2) (*n* = 407) n (%)	Williams and others (1991) [21] *n* (%)	Yentis & Lee (1998) [22] *n* (%)	Cook (2000) [23] *n* (%)	Suzuki and others (2012) [24] *n* (%)
I	186 (49.7)	228 (56.0)	1205 (86.9)	450 (67.9)	334 (66.8)	175 (54.7)
II	163 (43.6)	151 (37.1)	140 (10.1)	205 (30.9)	143 (28.6)	99 (30.9)
III	23 (6.1)	24 (5.9)	42 (3.0)	8 (1.2)	23 (4.6)	42 (13.1)
IV	2 (0.5)	2 (0.5)	0	0	0	4 (1.2)
Not Recorded	0	2 (0.5)

## Discussion

The main findings of this study are that the need for ancillary manoeuvres and the time taken for intubation are reduced when the patients are 25° back-up. The back-up position does not appear to improve glottic views in an unselected surgical population who were not anticipated to have difficult airways. Ninety-three percent of views obtained were classified as Cormack and Lehane Grades I or II; the median POGO score was 80.

The 25° back-up position was chosen as this incline was used by Lee and others [5] when they reported improved POGO scores at 25° in a prospective randomized cross-over trial in forty patients. In a cadaveric study, Levitan [14] suggested that increasing elevation of the head (relative to the horizontal) may reduce the required directional force along the laryngoscope handle and improve the operator’s line of sight down the laryngoscope blade. Lebowitz and others [11] concluded that shoulder and head elevation by any means that brings the patient’s sternum onto the horizontal plane of the external auditory meatus and maintains or improves laryngoscopic view significantly more often than it hinders it.

Our study attempted to help clarify whether the back-up position made intubation easier for the anaesthetist. Moving the operating table into the backup position is a simple inexpensive manoeuvre which may enable the line of sight fall more quickly upon the glottis. In Part 2, the combination of the sniffing and back-up position was not recorded as interfering with the insertion of the laryngoscope blade or decreasing the effectiveness of cricoid pressure or other laryngeal manoeuvres. A disadvantage of the back-up position is that lowering the operating table may not be sufficient to satisfactorily align the patient’s head at the level of a short anaesthetist’s sternum; a footstool may be required.

The shorter time taken for intubation when the patients are 25° back-up is relatively modest at 4 s. Both training and senior grades completed intubation faster in the back-up position and the difference although not significant was larger for the senior anaesthetists (Table 4). The distribution of intubation times is skewed to the left implying that it will always be more difficult to shave time off a procedure which is already quickly and proficiently carried out on most occasions. Four seconds is a 14 % reduction in intubation times. Viewed in this light (and the difference seconds can make as the patient slips down the oxygen dissociation curve) the shorter time may be a clinically useful safety advantage. The reduced need for laryngeal manoeuvres may be responsible for the shorter time as in clinical practice, laryngeal manipulation takes only a few seconds as the anaesthetic team obtains a clearer view of the glottic aperture.

Although intubation was faster, we failed to find improved Cormack and Lehane grades or higher POGO scores in the back-up position. There are several possible explanations for this. A simple non-interventional change such as altering the patient’s position might be expected to make relatively modest improvements to glottic view compared to more sophisticated aids such as advanced laryngoscopes. The back-up position improves the view most successfully when intubation is anticipated to be difficult, for example in obstetric or bariatric surgery; its impact when the patients’ airways are likely to be normal may well be less. Cormack and Lehane Grades I and II views and views with a POGO score above the median of 80 are satisfactory for successful intubation. If the back-up position produces only small improvements in glottic view during routine clinical practice where difficulties are unanticipated, a much larger study would be required. The present study was underpowered to detect small differences.

Figure 3 illustrates the problems of studying glottic views with both ordinal and continuous scales. Anaesthetists’ perceptions of how the POGO score corresponds to a Cormack and Lehane grade clearly vary. It is difficult to understand how POGO scores of less than 90 can also be classified as a Cormack and Lehane Grade I view and yet in both parts of the study 91 Grade I views were reported with POGO scores of less than 90. However, during intubation it may be difficult for the laryngoscopist to recall exactly what was seen without later verification of a recorded image. Inconsistency in application of the Cormack and Lehane grades is not new and has been highlighted previously [15, 16]; this study reinforces our specialty’s problems with reliably describing glottic views. Misclassification is likely to confound studies of glottic views and will have contributed to the debate about how best to describe what is seen when intubating. Although this study required the intubating anaesthetist to record the best view obtained, it is possible that the view recorded was actually an adequate view that allowed straight forward intubation in the clinical situation.

The pragmatic observational design of the study inevitably leads to a number of weaknesses. One of the most important was the number of anaesthetists contributing to the data. Although a total of 68 personnel contributed, the majority did not enrol more than 10 patients. This will have increased the inter-observer variability and may have been responsible for some misclassification. However, the ratio of training to permanent anaesthetic staff was similar in both parts of the study. Other weaknesses in the methodology include the inevitable unblinded nature of the study and the selection of patients. Unfortunately due to the relatively small number of recording laryngoscopes within the Department, independent verification of the glottic view was not possible. Adult Macintosh blades remain the most commonly used at Glan Clwyd Hospital. Although regularly encouraged, the decision to enrol patients was left to the individual anaesthetist; the number of patients who could have been enrolled but were not studied is unknown. Such variability combined with a lower incidence of Grade I views actually found rather than assumed in the original power calculation underpowered the study with regard to improvements in glottis views.

While randomised controlled trials are the gold standard for scientific investigation, the efficacy of such trials tends to decline when applied outside the original trial conditions because of the vagaries inevitably encountered in routine clinical practice, such as different operators and unselected surgical patients. The present study was deliberately designed to observe the effect of a simple non-interventional change in routine clinical practice. Despite its weaknesses, the present study’s results may be robust when suggesting that intubation is easier and quicker in the back-up position. Even though more nasal intubations and facio-maxillary procedures were included in the second phase, intubation time was shorter.

Patients predicted to have difficult airways were excluded from this study and so intubation was anticipated as ‘routine’. Recognising our inter-observer variability, our distribution of the Cormack and Lehane grades do not reflect the glottic views reported by Williams. There seems to have been a progressive decline in the completeness of glottis views over time (Table 5). In 2005, Shiga [17] reported the incidence of Cormack and Lehane Grades III and IV as 5.8 % in over 50,000 ‘normal’ patients which is similar to our results (i.e. 6.5 %). Reasons for this change are not clear but over the last 20 years, the surgical population as a subset of the general population has become more obese. In England between 1993 and 2012, the adults that were overweight/obese (body mass index 25.0 to >30) increased from 57.6 to 66.6 % in men and from 48.6 to 57.2 % in women [18]. In obese patients, back-up positioning has gas exchange, diaphragm positioning, and oxygenation advantages [19, 20]; easier intubation in the back-up position may enhance these safety advantages as the prevalence obesity increases in modern anaesthetic practice. However, the introduction of supraglottic airways has dispensed with the need to intubate patients for operations that would have previously mandated intubation. As a result the frequency of intubations for the individual anaesthetist may have declined over the decades resulting in inevitable skill loss. Further work is required to confirm whether views are indeed becoming less complete and explore the reasons behind this.

## Conclusion

In conclusion, this study has confirmed that when used at a departmental level, the 25° back-up position reduced the need for laryngeal manipulation and the time taken for intubation. The study was underpowered to determine whether or not glottic views were improved. In an unselected surgical population, the distribution of glottic views seems to be changing over time with less complete views becoming more frequent.

## Additional file

Additional file 1: Data Collection Form. (DOCX 189 kb)

## Abbreviations

IQR: Interquartile range
n: Number
POGO: Percentage of glottic opening
SD: Standard deviation
