# Supplementary material for: Comparison of glottic views and intubation times in the supine and 25 degree back-up positions
Source: BMC Anesthesiol. 2016 Nov 16;16:113. doi: 10.1186/s12871-016-0280-4 (PMC5112746; doi:10.1186/s12871-016-0280-4)
Supplement: Additional file 1: — Data Collection Form. (DOCX 189 kb) [file 12871_2016_280_MOESM1_ESM.docx]

Data Collection Form

***Glottic views in the classic sniffing position compared with the back-up position***

25 degrees

***Included:*** *All adult patients who require intubation as part of their anaesthesia*

***Excluded:*** *<18 years old*

*Known difficult airways where an alternative method of intubation (i.e. fibre optic) is the method of choice.*

*Patients undergoing emergency surgery where patient positioning and data collection might cause delay (e.g. exsanguinating patients) or where the supine position is not optimal (e.g. brisk bleeding into the upper airway)*

*Patients requiring rapid sequence induction of anaesthesia*

***Preconditions: Please tick***

| A head ring or non compressible pillow so that the head is raised 7 to 9 cm from the supine position |  |
| --- | --- |
| A line from the sternal notch to external auditory meatus is horizontal when in the supine sniffing position. |  |
| The back of the operating table is horizontal or raised by at least 25 degrees from the hip (depending upon phase of the study) |  |
| Nerve stimulator attached to ensure complete paralysis prior to laryngoscopy and intubation |  |

*Patient demographics*:

Age

Sex

Hospital number:

G number

Date

BMI and weight:

Surgery type (e.g. trauma, urology, general)

*Previous grade of laryngeal view* (if available from previous anaesthetic charts) n/a I II III IV

*All Drugs (and doses) Used*

|  |  |  |
| --- | --- | --- |
|  |  |  |
|  |  |  |
|  |  |  |

*View obtained*

The best view obtained during laryngoscopy reported by the anaesthetist performing the intubation.


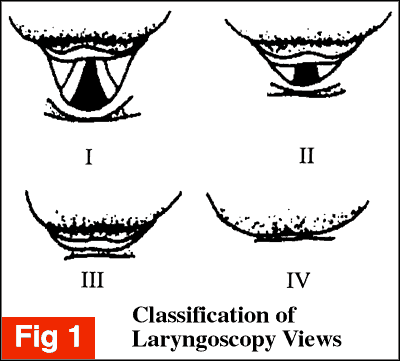

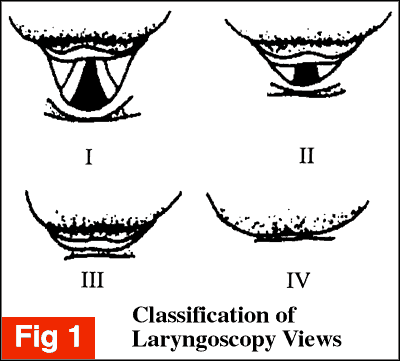

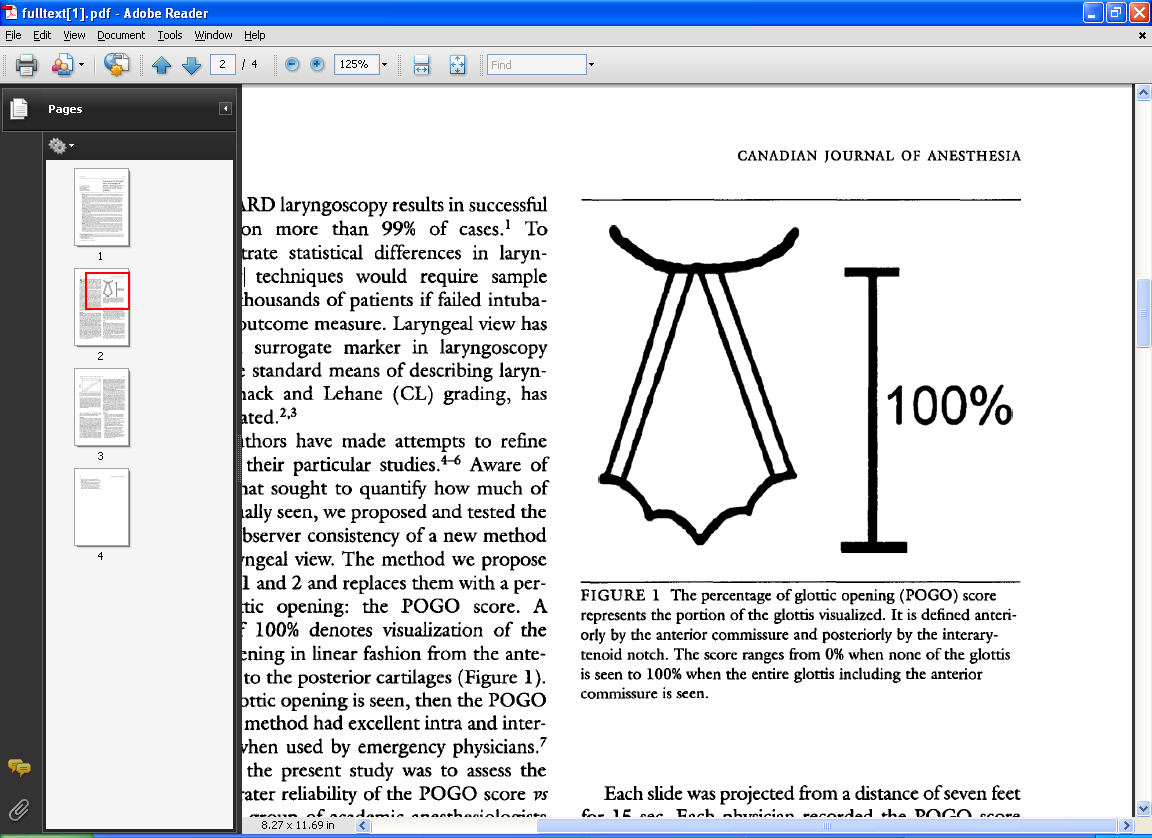


Cormack-Lehane:

POGO Score:

The percentage of glottic opening (POGO) score represents the portion of the glottis visualized. It is defined anteriorly by the anterior commissure and posteriorly by the interarytenoid notch. The score ranges from 0% when none of the glottis is seen to 100% when the entire glottis including the anterior commissure is seen..

| *Number of attempts* at laryngoscopy and tracheal intubation. | *Laryngoscopy:*  *Tracheal intubation:*  *Nasal or Oral:* |
| --- | --- |
| *Use of ancillary equipment* (e.g. bougie) |  |
| *Use of ancillary manoeuvres* (e.g. laryngeal manipulation) |  |
|  |  |
| The time (mm:sec) between the loss of consciousness (loss of eyelash reflex or loss of verbal response) and detection of CO2 on the end-tidal CO2 monitor after the successful placement of the tracheal tube |  |
| The time (mm:sec) from beginning of laryngoscopy to insertion of the tracheal tube (time of intubation) |  |
|  |  |
| Damage to adjacent tissues (e.g. lips or teeth) |  |
| Additional notes |  |

Name of anaesthetist: Grade: Years of experience:

Please return to Anaesthetic Dept, YGC
